# The Course of Epithelioma

**Published:** 1871-08

**Authors:** 


### ARTICLE XI.

### *The Course of Epithelioma.*

Dr. Reid, in the *Glasgow Medical Journal*, writes as follows:

In accordance with this view, the development and course of the disease would be somewhat in the following order:—

1. An increase of epithelial cells, with more or less irritation and hyperæmia of the neighboring parts. The increase of epithelial cells, and consequently hypertrophy of the glands in this variety—the sub-cutaneous—takes place at the expense of the connective tissues, which are partly displaced and condensed, and partly incorporated with the gland tissue.

2. A proliferation and degeneration of the nuclei increasing the volume, and at the same time obscuring the original glandular type. The cuticle yields to the pressure exerted by the softened and partially disintegrated mass which is discharged in the form of brownish crusts. The ulcerated surface may either imperfectly cicatrize or remain as an open sore covered with a loose scab. The part discharged is soon replaced by others. In this way there is a succession of discharges at a shorter or longer interval, the ultimate result being an extension of the ulcer. Whilst this process is going on in the centre, new elements are added to the circumference, and the area of the disease extends with that of the ulcer. At this early stage the aggregation of hypertrophied glands resembles a small well defined tumor, which in the later stages is represented by the cancroid deposit at the margin of the ulcerated surface. The imperfect cicatrization of the early stages and the permanently ulcerated surface in the later stage, covered with a brownish crust, appear to be due to the infiltration of the connective tissues with epithelial and other elements.

